# Recurrent adamantinomatous craniopharyngiomas show MAPK pathway activation, clonal evolution and rare *TP53*-loss-mediated malignant progression

**DOI:** 10.1186/s40478-024-01838-4

**Published:** 2024-08-10

**Authors:** John R. Apps, Jose Mario Gonzalez-Meljem, Romain Guiho, Jessica C. Pickles, Eric Prince, Edward Schwalbe, Nikhil Joshi, Thomas J. Stone, Olumide Ogunbiyi, Jane Chalker, Akang Bassey, Georg Otto, Rosalind Davies, Debbie Hughes, Sebastian Brandner, Enrica Tan, Victoria Lee, Caroline Hayhurst, Cassie Kline, Sergi Castellano, Todd Hankinson, Timo Deutschbein, Thomas S. Jacques, Juan Pedro Martinez-Barbera

**Affiliations:** 1 Institute of Cancer and Genomic Sciences, Edgbaston Campus, University of Birmingham, Birmingham, B15 2TT UK; 2 Developmental Biology and Cancer Research and Teaching Department, UCL Great Ormond Street Institute of Child Health, University College London, London, UK; 3 Birmingham Women’s and Children’s NHS Foundation Trust, Birmingham, UK; 4 Tecnologico de Monterrey, School of Engineering and Sciences, Mexico City, Mexico; 5 Oniris, INSERM, Regenerative Medicine and Skeleton, RMeS, UMR 1229, Nantes Université, 44000 Nantes, France; 6 Departments of Neurosurgery and Pediatrics, Children’s Hospital Colorado, University of Colorado School of Medicine, Aurora, USA; 7 Department of Applied Sciences, Faculty of Health and Life Sciences, Northumbria University, Newcastle upon Tyne, UK; 8 Division of Oncology, Department of Pediatrics, Children’s Hospital of Philadelphia, Philadelphia, USA; 9 Great Ormond Street Hospital for Children, NHS Foundation Trust, London, UK; 10 Institute of Cancer Research, Sutton, UK; 11 Department of Neurodegenerative Disease, UCL Queen Square Institute of Neurology, University College London, London, UK; 12 Division of Neuropathology, National Hospital for Neurology and Neurosurgery, University College London Hospitals NHS Foundation Trust, London, UK; 13 KK Women’s and Children’s Hospital, Singapore, Singapore; 14 Sheffield Children’s Hospital NHS Foundation Trust, Sheffield, UK; 15 University Hospital of Wales, Cardiff, UK; 16 Perelman School of Medicine, University of Pennsylvania, Philadelphia, PA 19104 USA; 17 Department of Internal Medicine I, Division of Endocrinology and Diabetes, University Hospital, University of Würzburg, Würzburg, Germany; 18 Medicover Oldenburg MVZ, Oldenburg, Germany

**Keywords:** Craniopharyngioma, MAPK signalling pathway, MEK inhibitor, Macrophage/microglia

## Abstract

The two types of craniopharyngioma, adamantinomatous (ACP) and papillary (PCP), are clinically relevant tumours in children and adults. Although the biology of primary craniopharyngioma is starting to be unravelled, little is known about the biology of recurrence. To fill this gap in knowledge, we have analysed through methylation array, RNA sequencing and pERK1/2 immunohistochemistry a cohort of paired primary and recurrent samples (32 samples from 14 cases of ACP and 4 cases of PCP). We show the presence of copy number alterations and clonal evolution across recurrence in 6 cases of ACP, and analysis of additional whole genome sequencing data from the Children’s Brain Tumour Network confirms chromosomal arm copy number changes in at least 7/67 ACP cases. The activation of the MAPK/ERK pathway, a feature previously shown in primary ACP, is observed in all but one recurrent cases of ACP. The only ACP without MAPK activation is an aggressive case of recurrent malignant human craniopharyngioma harbouring a *CTNNB1* mutation and loss of *TP53*. Providing support for a functional role of this *TP53* mutation, we show that *Trp53* loss in a murine model of ACP results in aggressive tumours and reduced mouse survival. Finally, we characterise the tumour immune infiltrate showing differences in the cellular composition and spatial distribution between ACP and PCP. Together, these analyses have revealed novel insights into recurrent craniopharyngioma and provided preclinical evidence supporting the evaluation of MAPK pathway inhibitors and immunomodulatory approaches in clinical trials in against recurrent ACP.

**Supplementary Information:**

The online version contains supplementary material available at 10.1186/s40478-024-01838-4.

## Introduction

Craniopharyngiomas are clinically challenging tumours of the sellar region associated with significant morbidity and increased late mortality [1–4]. There are two types, adamantinomatous (ACP), seen in children and adults and characterised by exon 3 mutations in *CTNNB1* and papillary (PCP), predominantly seen in adults and characterised by *BRAF* V600E mutations [3]. No additional recurrent mutations or genomic changes have been identified across these tumour types so far, with just one report suggesting limited recurrent focal gains or losses (Xp28) in a subset of ACP patients [5].

Recurrence/regrowth of craniopharyngioma is seen in approximately 25% of cases, despite either gross surgical resection or incomplete resection with adjuvant radiotherapy [6, 7]. In a subset of ACP patients, recurrence occurs frequently, posing clinical management challenges and highlighting the need to identify novel therapies [8, 9]. In addition, malignant transformation of ACP has been described in a rare subset of cases in the literature, usually in association with previous radiotherapy and/or frequent tumour recurrences [10–13]. In those cases, where pathology has been assessed, malignant tumours have shown high Ki67 and p53 expression [12, 13]. Despite the clinical relevance, the biological processes underlying tumour recurrence or malignant transformation of ACP remain mostly unknown [2, 4].

The MAPK/ERK signalling pathway is activated in PCP as a consequence of the oncogenic *p.BRAF-V600E* variant present in these tumours, and therapeutic inhibition of this pathway is showing very promising results in various human studies [14–20]. ACPs do not carry mutations in components of the MAPK/ERK pathway, however, this pathway is activated in a paracrine manner due to the expression of multiple secreted factors such as members of the FGF protein family (e.g. FGF3/4/9), EGF as well as other ligands and transmembrane proteins capable of activating the pathway (e.g., CD47) [21–23]. Preclinical experiments in explant cultures of human and murine (*Hesx1*^*Cre/*+^; *Ctnnb1*^*lox(ex3)/*+^ mouse model) ACP tumours have shown that treatment with Trametinib, a clinically approved MEK inhibitor, can reduce proliferation and induce apoptosis in ACP tumour cells [22]. Likewise, inhibition of the pathway using an ERK1/2 inhibitor in human ACP cell cultures results in reduced proliferation and invasion [21]. Building on these initial observations, the MEK inhibitor Binimetinib has been used in a patient with an aggressive ACP, who showed a measurable radiological response [9]. Clinical trials are now underway to evaluate the role of MAPK inhibition in ACP (NCT05286788, NCT05465174). However, little has been described on the activation of the MAPK pathway in recurrent ACP tumours. This is relevant to support the inclusion of patients with recurrent ACP in the ongoing clinical trials.

High levels of inflammatory mediators (e.g., IL6, IL8, IL1) have been observed in ACP in both the cystic and solid components, and targeting with the IL6 inhibitor Tocilizumab has shown modest responses in cystic tumours and is under evaluation in clinical trials (NCT05233397) [8, 22, 24]. Likewise, immune checkpoint proteins such as PD1, PDL1 and CD47 have been identified in ACP tumours transcriptionally and immunohistochemically, and suggested as therapeutic targets, with one clinical trial combining anti-PD1 therapy with MAPK pathway (RAF) inhibition (NCT05465174) [8, 22, 24–29]. Fewer studies have interrogated the immune microenvironment of PCP to date, but those studies have also revealed a complex environment with pro-inflammatory and anti-inflammatory components [30].

In this manuscript, we explore the biology of recurrence craniopharyngioma in a cohort of recurrent ACP and PCP tumours and validate findings across independent cohorts, datasets and through the use of existing mouse models of craniopharyngioma, with both pre-clinical drug testing and further genetic mouse modelling.

## Materials and methods

### Human samples

FFPE tissue from ACP and PCP was obtained through local archives, the Children’s Cancer and Leukaemia Group tissue bank, Brain UK [31] and from international partners. All work was within ethical approval REC14/LO/2265, REC19/SC/0217 and REC21/L0/0707. Tumours were prioritised where there was matched primary and relapse or sequential relapse material available or particularly aggressive tumours (i.e., requiring multiple serial operations, frequent regrowth or malignant histology). Importantly, we used tumours in which the proportions of epithelial tumour and glial reactive tissue were similar across the serial samples to avoid bias caused by comparing tumour samples of different cell compositions, a particular challenge in this tumour type, where there can be considerable variability between tumours [22]. A control group of non-relapsing tumours was included. In total 32 samples from 14 cases of ACP and 4 cases of PCP, were analysed. Details are summarised in Additional Table 1.

Epithelial tumour content was assessed by H&E from sections immediately before and after the sections used or RNA and DNA isolation. Two 10 µm FFPE histological sections were used for RNA extractions and two further 10 µm sections for DNA extraction. Macro-dissection to remove non-tumour tissue was used in ACP14, due to heterogeneity across the section, to reduce areas of low tumour content. *CTNNB1* and *BRAF* variant status was confirmed in all cases where data was available, either from evaluation during standard-of-care clinical analysis, panel sequencing, or RNA sequencing (see below) (Additional Tables 1 and 2).

### DNA methylation analysis and RNA sequencing

DNA was extracted using the Maxwell 16 FFPE Tissue LEV DNA Purification Kit on a Maxwell 16 Research Instrument (Promega, USA) according to the manufacturer’s instructions. 250 ng eluted DNA was subjected to bisulphite conversion using the Zymo EZ DNA Methylation-Gold Kit (Zymo Research, USA). Bisulphite-converted DNA was additionally treated using the Infinium FFPE DNA restore Kit prior to assay on the Illumina HumanMethylationEPIC BeadChip platform v1.0 (Illumina, USA), in accordance with the Infinium HD Assay protocol. Processed arrays were scanned using an Illumina IScan array scanner to generate IDAT output files. For RNA extraction and sequencing, sections were deparaffinised using Qiagen deparaffinisation solution (Cat No./ID: 19093) and RNA was extracted using Qiagen miRNeasy FFPE kit (Cat No./ID: 217504) with DNAse I treatment, as per manufacturer’s instructions, and eluted into 30ul ddH20. RNAs were quantified using Agilent Tapestation, and samples with DV200 > 20% were taken forward for library preparation using Agilent SureSelect XT RNA Library Prep, and sequenced on an Illumina HiSeq3000 (75 bp PE, dual 8 bp index, ~ 52 M reads/sample.

Panel sequencing was performed in a limited number of cases using 120 genes/regions of interest from an in-house paediatric solid tumour panel using Roche DNA capture and Illumina NovaSeq by the Clinical Genomics Translational Research group at the Royal Marsden Hospital [32]. The primary analysis was performed using Molecular Diagnostics Information Management System v4.0, based on genome build hg19.

Due to limited quantities of material in some of the samples, and variable success of nucleotide extraction and sequencing, not all samples had successful methylation and RNAseq data available. Full details of samples and analyses can be found in Additional Table 2. Bioinformatic analyses are described in Additional file 1.

### Analysis of external datasets

Publicly available copy number data derived from whole genome sequencing (WGS) data of 101 craniopharyngiomas from 91 patients was downloaded from the Children’s Brain Tumour Network (CBTN), a large open-access cohort of brain tumour cases from multiple institutions [33] (https://cbtn.org/). Chromosomal arm-level changes were scored from segmented copy number data as previously described [34]. To ensure that cases in which copy number alterations had been identified were from ACP tumour samples with high epithelial tumour content, we included only those cases with a confirmed *CTNNB1* mutation (70 samples from 67 cases). The recently published ACP single-cell and single-nucleus RNA sequencing dataset (sc/snRNA-seq) was used to further interrogate the immune microenvironment of ACP [35]. Bioinformatic analyses are described in Additional file 1.

### Immunohistochemistry

All immunohistochemistry staining on human tissue was done on Leica Bondmax and histological slides were digitised to whole slide images and scanned on either a Hamamatsu NanoZoomer S360 or Aperio CS2 Scanner (S/N 5872) at 40 × final resolution or 0.25 µm/pixel. Immunohistochemistry on mouse tissue was performed as previously described [36]. Visualization of mouse sample H&E and immunohistochemistry staining was conducted in a Zeiss Axioplan2 microscope and captured with a Zeiss Axiocam HRc colour camera. Double immunofluorescence was performed as previously described [22]. Immunofluorescent staining in both human and murine samples was visualized with a Leica DMLM widefield microscope and imaged with a CoolSnap monochrome camera or with a Zeiss Axio Observer with a Hamamatsu ORCA camera. Image processing was conducted using Fiji/ImageJ, which included brightness/contrast enhancement and merging of fluorescence channels to produce composite images. Details of antibody and epitope retrieval can be found within Additional file 1.

### Ex vivo culture of murine neoplastic pituitaries and drug treatments

Neoplastic pituitaries from 18.5dpc *Hesx1*^Cre/+^;*Ctnnb1*^*lox(ex3)/*+^ embryos [37, 38] were dissected and placed on 5 μM Nucleopore hydrophilic membranes (VWR) in 24 well plates containing 500 μl of medium (DMEM-F12, Gibco, 1% Pen/Strep, Sigma and 1% FBS, Thermo Fisher Scientific). Cultures were treated with either Selumetinib (ApexBio, 50 nM) or Binimetinib (Cayman Chem., 500 nM), or vehicle (0.1% DMSO) for 16–24 h and subsequently fixed and processed for histological analysis. Immunofluorescence staining was performed as previously described [36]. The proportion of Ki-67 positive cells was calculated as an index out of the total DAPI-stained nuclei. Over 170,000 DAPI nuclei were counted from 10 to 23 histological sections per condition, in a total of nine neoplastic pituitaries using Fiji/ImageJ [39]. Due to the high tissue density, the proportion of cytoplasmic markers cleaved-caspase-3 and pERK1/2 positive cells were calculated as an index out of the total tissue area, from 9 to 21 histological sections per condition.

### Generation and analysis of a mouse ACP model carrying inactivating *Trp53* mutations

*Hesx1*^*Cre/*+^ mice have previously been characterised and shown to express the recombinase Cre in the early precursors of the anterior pituitary [40]. *Ctnnb1*^*lox(ex3)/*+^ mice contain *loxP* sites flanking exon 3 of the *Ctnnb1* gene [41]. Cre expression leads to exons 2 and 4 being connected in-frame, producing a degradation-resistant and transcriptionally functional β-catenin protein missing the amino acids encoded by exon 3. *Trp53*^*fl/fl*^ mice have also been previously described [42] and contain *loxP* sequences flanking exons 2 and 10, which effectively produce a null mutation upon Cre-mediated deletion. *Trp53*^*fl/fl*^ mice were crossed with *Hesx1*^*Cre/*+^ and *Ctnnb1*^*lox(ex3)/lox(ex3)*^ mice to produce*: Hesx1*^*Cre/*+^; *Ctnnb1*^*lox(ex3)/*+^; *Trp53*^*fl/*+^ controls and *Hesx1*^*Cre/*+^; *Ctnnb1*^*lox(ex3)/*+^; *Trp53*^*fl/fl*^ experimental mice.

For the survival study, age-matched males and females, of the following genotypes were used: *Hesx1*^*Cre/*+^; *Ctnnb1*^*lox(ex3)/*+^; *Trp53*^*fl/fl*^, *Hesx1*^*Cre/*+^; *Ctnnb1*^*lox(ex3)/*+^; *Trp53*^*fl/*+^ and* Hesx1*^*Cre/*+^; *Trp53*^*fl/fl*^*.* A humane end-point was determined in line with UK Home Office regulation regarding the use of mice in research. Death was not used as a humane end-point, instead, mice were humanely culled when health deterioration was assessed to be irreversible. Mice were kept in intra-ventilated cages, with free access to food and water and maintained in a 12-h light–dark cycle. End-point tumours were collected and further dissected in ice-cold Dulbecco’s Modified Eagle’s Medium supplemented with 10% Fetal Calf Serum (FCS). Further details of phenotyping are presented in Additional file 1.

## Results

### DNA methylation profiling identifies genomic evolution and acquisition of copy number changes in ACP

**Fig. 1:**
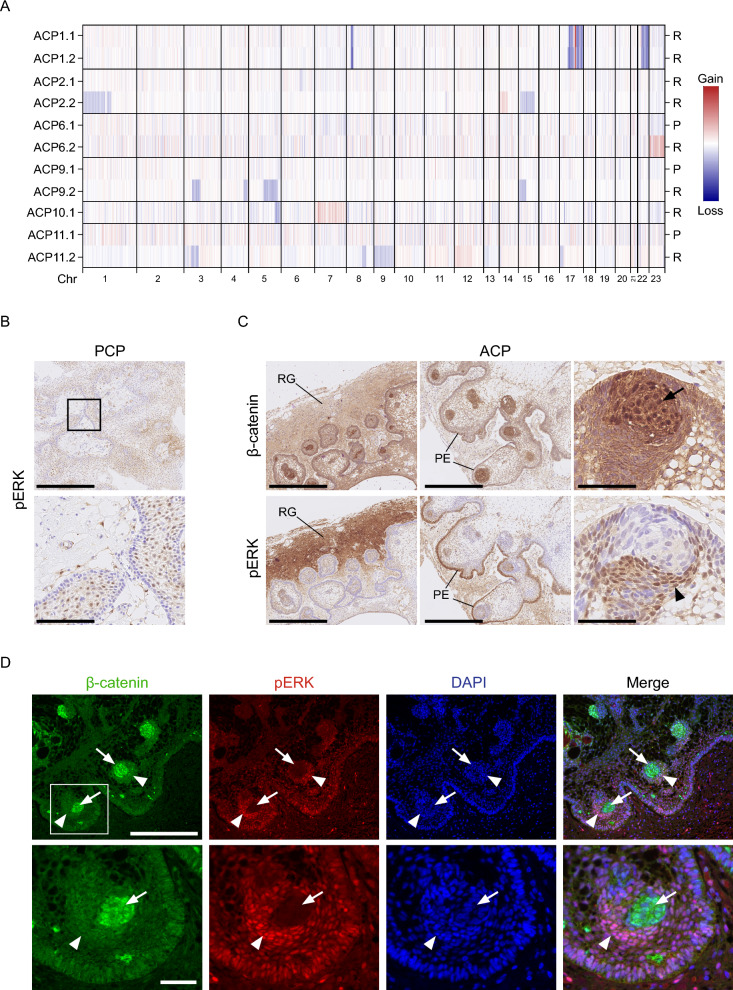
Genomic evolution and MAPK pathway activation in craniopharyngioma. **A** Heatmap showing copy number changes identified in recurrent craniopharyngioma samples. Red indicates gain, blue indicates loss. P indicates primary tumour, R indicates recurrence. In 6 cases, copy number alterations were identified. Specifically,in two recurrence samples of case 1, and in cases 2, 6, 9, 10, 11, where changes were only identified in the most recent recurrences. ACP2.1, 9.1 and 11.1 are primary samples, all others are from tumour recurrences. Full details of each sample are in Additional Tables 1 and 2. **B** pERK1/2 immunohistochemistry of PCP showing activation restricted to a supra-basal layer. Scale bars: 500 µm for low power image; 100 µm for high power image (inset). **C** β-catenin (top row) and pERK1/2 (bottom row) immunohistochemistry in consecutive ACP sections showing pERK1/2 activation in reactive glia (RG, left column), palisading epithelia (PE, middle column) and pERK1/2-positive cells (arrowhead) surrounding a nuclear β-catenin accumulating cluster (arrow, right column) (3 different ACP cases are shown). Scale bars: 500 µm for left and middle columns; 50 µm for right column. **D** Double immunofluorescence staining confirming the close relation, but mutual exclusivity of pERK1/2-positive cells (arrowheads) with nucleo-cytoplasmic β-catenin accumulating clusters (arrows). Scale bars: 500 µm for first row; 100 µm for second row (inset)

Methylation analysis was performed on 22 samples from 13 ACP patients and on 5 samples from 3 PCP patients (Additional Table 2). Analysis of chromosomal copy number plots identified whole chromosomal or segmental chromosomal copy number variations (CNVs) in the recurrent samples from six cases of ACP (ACP1,2,6,9,10,11) (Fig. 1A, Additional Fig. 1A). None of the primary tumours for which data were available (ACP6,9,11) showed any CNVs. In ACP1, CNVs were conserved across two recurrences (Fig. 1A). However, CNVs were identified only in the most recent recurrence in ACP2, but not in a previous recurrence (Fig. 1A). Together, these observations suggest that at least a proportion of ACPs undergo genomic evolution, as indicated by the acquisition of CNVs, as they progress from primary to recurrent tumours. Importantly in at least two of these cases radiotherapy had not been administered. Further details of cases and analysis are found in Additional Tables 1 and 2.

To further explore the presence of copy number changes in craniopharyngioma, we explored a WGS dataset of 70 samples from 67 cases with confirmed *CTNNB1* mutation, and/or methylation classification as ACP from CBTN [33]. This identified 7 cases (7/67 (10%)) with chromosomal arm copy number changes (Additional Fig. 1B, Additional Table 3). Clinical data showed five of these samples were from a primary resection, and two from recurrences, showing that copy number changes can occur at any stage and are not dependent on therapy. There were no serial samples available for cases with copy number changes, so it was not possible to further explore clonal evolution in this cohort.

In summary, these analyses confirm that large-scale copy number changes are observed in a proportion of ACP, both at primary resection and recurrence, and that clonal evolution can occur across recurrence.

### MAPK pathway activation is observed in primary and recurrent ACP and PCP tumours

Activation of the MAPK/ERK pathway has been studied mostly in primary craniopharyngioma, despite clinical trials now underway targeting this pathway in patients with relapsed PCP (NCT03224767) and ACP (NCT05286788, NCT05465174). We therefore explored the activation of the MAPK pathway in this cohort of primary and recurrent craniopharyngiomas, by immunohistochemistry against phosphorylated (p)-ERK1/2, a read-out of the activated pathway [22].

In PCP tumours, p-ERK1/2 staining was restricted to cells in a supra-basal layer lining the fibrovascular cores, as previously described [43] and was detected in all primary and recurrent tumours (n = 7) (Additional Table 2; Fig. 1B). In ACP tumours, p-ERK1/2 staining was heterogeneous within samples and was predominantly observed in the palisading epithelium and reactive glial tissue adjacent to it (i.e., the invasive front), although not all areas of palisading epithelium were positive (Fig. 1C; Additional Fig. 2; Additional Table 2). Additionally, p-ERK1/2 staining was also often detected in cells in close proximity with the β-catenin-accumulating clusters (Fig. 1C), but not in the cluster cells (Fig. 1D). This pattern of expression of p-ERK1/2 was observed in all primary tumours analysed (n = 6) and 6 of 7 recurrent tumours (Additional Fig. 2). The one ACP recurrent tumour that did not show this typical p-ERK1/2 staining was a malignant case (ACP11) (see below). These data indicate that the activation of the MAPK/ERK pathway is a highly conserved feature in both primary and recurrent PCP and ACP.

**Fig. 2:**
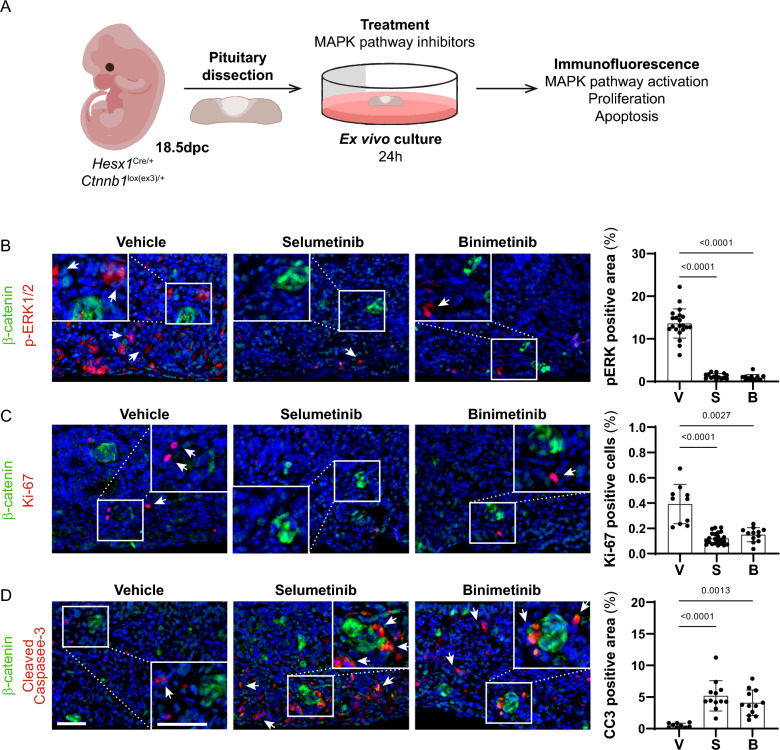
Selumetinib and Binimetinib reduce proliferation and induce apoptosis in explants cultures of murine ACP. **A** Experimental design: Tumoural pituitaries from 18.5 dpc *Hesx1*^Cre/+^; *Ctnnb1*^lox(ex3)/+^ embryos were dissected and cultured in the presence of Selumetinib (50 nM; n = 3), Binimetinib (500 nM; n = 3) or vehicle (DMSO 0.1%; n = 3), and processed for histological analysis after 24 h. **B** Immunofluorescence staining against β-catenin (green) and pERK1/2 (red), and quantification of pERK1/2 positive area show the inhibition of MAPK pathway after treatment with Selumetinib (S) or Binimetinib (B) compared with the vehicle control cultures (V). Note the pERK1/2 staining around the β-catenin accumulating clusters (arrows). **C** Immunofluorescence staining against β-catenin (green) and Ki-67 (red), and quantification of Ki-67 index as a percentage of total DAPI positive nuclei, show the proliferation inhibition after treatment with Selumetinib (S) or Binimetinib (B) compared with the vehicle control cultures (V). Note the presence of proliferative cells near the β-catenin accumulating clusters (arrows). **D** Immunofluorescence staining against β-catenin (green) and cleaved Caspase-3 (CC3, red), and quantification of CC3 positive area, show the increase of apoptosis after treatment with Selumetinib (S) or Binimetinib (B) compared with the vehicle control cultures (V). Note the presence of apoptotic cells near β-catenin accumulating clusters and within tumour parenchyme (arrows). Main scale bar, 50 μm—Inset scale bar, 40 μm. *P*-values calculated with Kruskal–Wallis statistical test, followed by Dunn's multiple comparisons post-test

### Selumetinib and Binimetinib inhibit proliferation and increase apoptosis in explant cultures of murine ACP

Binimetinib and Selumetinib, have not been tested preclinically in ACP. Tumoural pituitaries of the *Hesx1*^*Cre/*+^; *Ctnnb1*^*lox(ex3)/*+^ mouse model [44] were cultured in explant cultures for 16–24 h in the presence of either 50 nM Selumetinib, 0.5 mM Binimetinib or vehicle as control, and processed for histological analysis (Fig. 2A). This time was chosen to assess the acute response to the inhibitor and to minimise cell death caused by prolonged culture [22]. These drug concentrations have been shown to be pharmacologically achievable in humans [45, 46]. Immunofluorescence staining against p-ERK1/2 confirmed the inhibition of the MAPK pathway in tumours treated with the MEK inhibitors relative to the controls (*p* < 0.001) (Fig. 2B). MAPK pathway inhibition was associated with a significant decrease in Ki67 and a significant increase in cleaved caspase-3 expression (marker of apoptosis) relative to the vehicle controls (*p* < 0.001) (Fig. 2C, D). Reduced proliferation and increased apoptosis are also observed when using the MEKi Trametinib in explant cultures of mouse and human ACP [22]. Together, these data confirm that Selumetinib and Binimetinib are suitable drugs to achieve the inhibition of the MAPK pathway in ACP and are appropriate for further evaluation in clinical trials.

### Altered β-catenin and p-ERK1/2 expression in a case of malignant craniopharyngioma with deletion of *TP53*

**Fig. 3:**
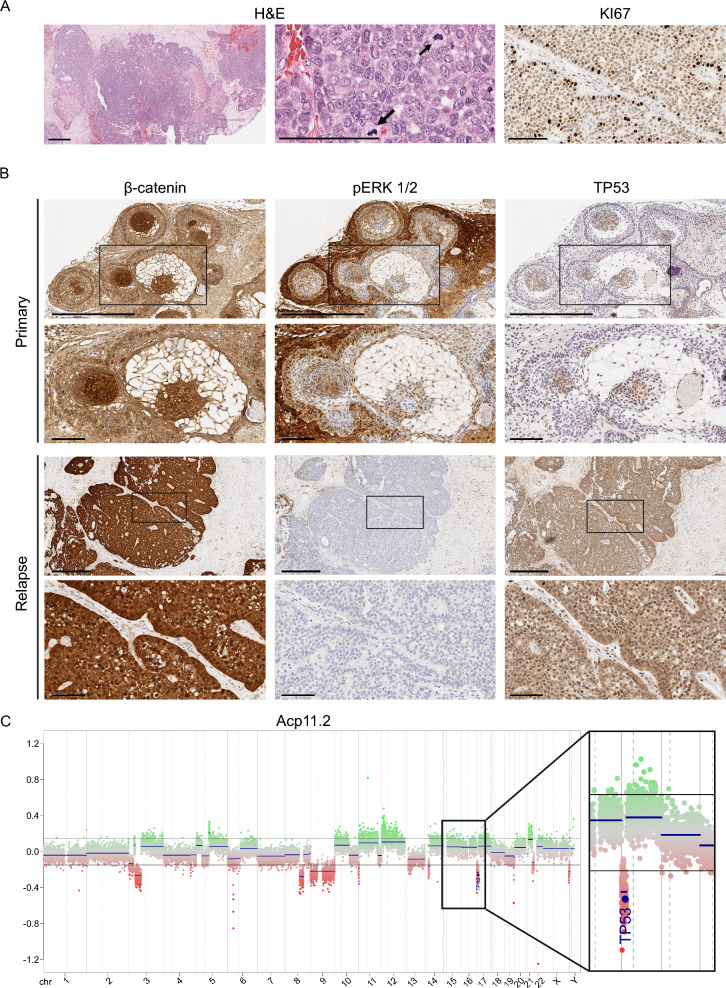
Altered distribution of β-catenin and paucity of pERK1/2 staining in a case of malignant craniopharyngioma with a heterozygous deletion of *TP53*. **A** Haematoxylin and eosin staining (left and middle images) and Ki67 immunohistochemistry (right image) at recurrence showing a poorly differentiated epithelial tumour with frequent mitoses. Scale bars: 500 μm for left image and 100 μm for middle and right images. **B** β-catenin, pERK1/2 and *TP53* immunohistochemistry (left, middle and right columns, respectively) showing altered distribution between primary (upper panel) and recurrent tumours (lower panel). Second row of each primary and recurrent panels show high magnification images indicated by insets. At recurrence the tumour has widespread nuclear β-catenin staining, *TP53* expression and loss of pERK1/2, **C** Copy number plot at recurrence showing multiple copy number changes, including deletion of *TP53*. Scale bars lower power 500 μm and higher power 100 μm

The primary ACP11 tumour showed classic features of ACP (palisading epithelia, wet keratin, epithelial whorls), however, histological analysis of this patient’s second recurrence revealed the presence of malignant features, including: (1) Poorly differentiated solid epithelial tumour composed of a mixture of solid nests, tubules and thin trabeculae infiltrating the stroma; (2) Frequent mitotic Figures (12/mm^2^); (3) KI67 positivity in the majority of the tumour cells (Fig. 3A). Wet keratin could be observed in the recurrent tumour. Immunohistochemistry against β-catenin in the primary tumour showed the typical staining, i.e., nucleo-cytoplasmic accumulation only in a minority of tumour cells, either individually dispersed or forming cell clusters, while the majority of the cells showed membranous β-catenin staining (Fig. 3B). In contrast, at recurrence, the vast majority of the tumour cells showed nucleo-cytoplasmic accumulation of β-catenin, with few cells showing membranous localisation (Fig. 3B). Importantly, a p.*CTNNB1-*G34R mutation was confirmed in both primary and recurrent tumours, confirming the clonal relationship between these samples (Additional Table 2). Moreover, although the primary tumour failed to classify on the methylation array using the DKFZ methylation classifier, possibly due to poor tissue quality or technical problems, the recurrence did classify as ACP (calibrated score = 0.998876) (Additional Table 2).

Examination of the copy number profile of the relapsed sample of ACP11 relative to the primary tumour sample revealed several CNVs including the acquisition of a deletion of *TP53* (Fig. 3C), which was further confirmed as heterozygous by next-generation sequencing. Moreover, *TP53* (p53) immunohistochemistry showed an abundance of cells with nuclear p53 staining in the recurrent, compared with the primary tumour (Fig. 3B). Strikingly, staining for p-ERK1/2 also revealed a change in the expression pattern. The primary tumour showed the typical expression pattern previously described (Fig. 3B; Additional Fig. 2) [21, 22]. In contrast, there was only minimal p-ERK1/2 staining in the relapsed sample, localised to a single small area close to the periphery of the tumour, suggesting that tumour cells do not express pERK1/2. These analyses demonstrate the identification of a *bona fide* malignant craniopharyngioma showing clear malignant histological transformation, preserved exon 3 *CTNNB1* mutation and heterozygous loss of *TP53* variant.

### *Trp53* deletion in a mouse model of craniopharyngioma leads to aggressive tumours with altered p-ERK/2 expression

**Fig. 4:**
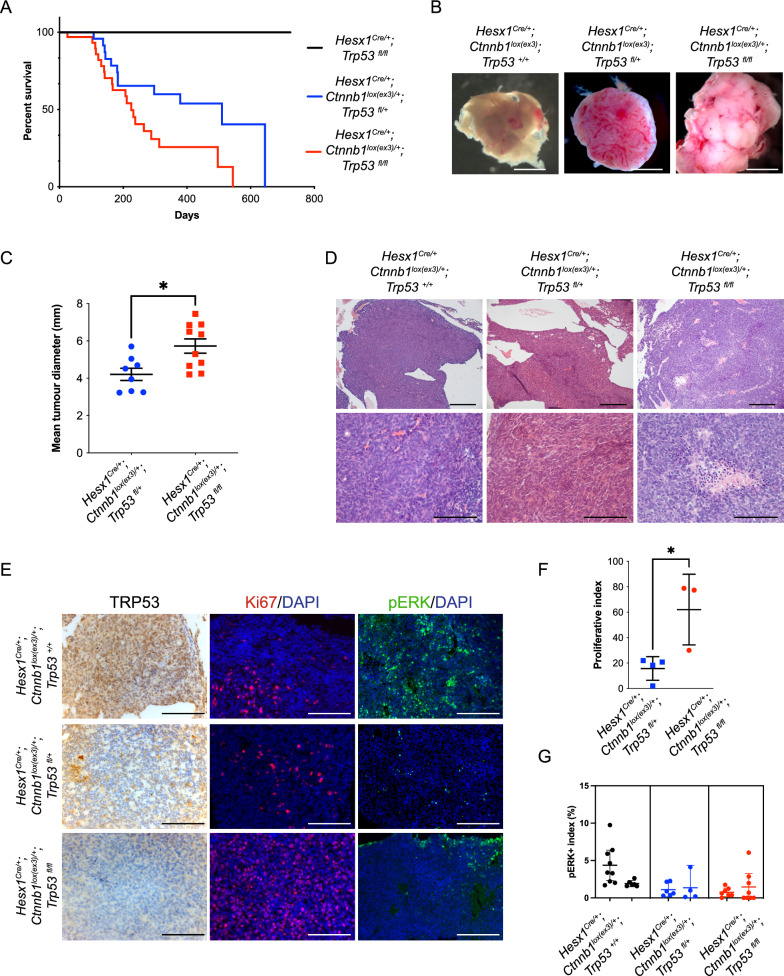
Deletion of *Trp53* in an ACP murine model results in increased tumour growth and reduced mouse survival. **A** Kaplan–Meier survival curve for *Hesx1*^*Cre/*+^; *Ctnnb1*^*lox(ex3)/*+^; *Trp53*^*fl/fl*^; mice (red line), *Hesx1*^*Cre/*+^; *Ctnnb1*^*lox(ex3)/*+^; *Trp53*^*fl/*+^ (blue line) and *Hesx1*^*Cre/*+^; *Trp53*^*fl/fl*^ mice (black line). Statistical comparison between *Hesx1*^*Cre/*+^; *Ctnnb1*^*lox(ex3)/*+^; *Trp53*^*fl/*+^ (n = 33) and *Hesx1*^*Cre/*+^; *Trp53*^*fl/fl*^ (n = 25) survival curves was conducted by a log-rank Mantel-Cox test (*P* = 0.0219). The mean survival is significantly reduced in mice lacking both *Trp53* alleles. **B** Representative images of tumours dissected at a humane endpoint showing the increased size found in the *Hesx1*^*Cre/*+^; *Ctnnb1*^*lox(ex3)/*+^; *Trp53*^*fl/fl*^; tumours. **C** Plot of mean tumour diameters showing a significant increase in *Trp53*-null (n = 10) versus *Trp53*-heterozygous (n = 8) genotypes (*P* = 0.0101**,** unpaired t test). Horizonal lines represent the mean and standard deviation. **D** Haematoxylin/eosin (H&E) staining of representative mouse ACP specimens. *Hesx1*^*Cre/*+^; *Ctnnb1*^*lox(ex3)/*+^; tumours (first column) are characterized by the presence of large cysts (top row) and a solid content formed by poorly differentiated non-epithelial cells. *Hesx1*^*Cre/*+^; *Ctnnb1*^*lox(ex3)/*+^; *Trp53*^*fl/*+^ tumours (middle column) display a similar histology. *Hesx1*^*Cre/*+^; *Ctnnb1*^*lox(ex3)/*+^; *Trp53*^*fl/fl*^; tumours (last column) show a distinct histologic phenotype characterized by a larger solid tumour content (top row) as well as higher number of mitotic bodies and necrotic regions (bottom row). **E** Molecular analysis by immunohistochemistry (IHC) and immunofluorescence (IF) of mouse ACP samples. Left column: IHC against TRP53 showing complete absence of P53 protein expression in *Hesx1*^*Cre/*+^; *Ctnnb1*^*lox(ex3)/*+^; *Trp53*^*fl/fl*^; tumours (bottom row) in contrast to *Hesx1*^*Cre/*+^; *Ctnnb1*^*lox(ex3)/*+^ and *Hesx1*^*Cre/*+^; *Ctnnb1*^*lox(ex3)/*+^; *Trp53*^*fl/*+^ tumours (top and middle rows respectively). Middle column: IF against KI67 shows increased cell proliferation in *Hesx1*^*Cre/*+^; *Ctnnb1*^*lox(ex3)/*+^; *Trp53*^*fl/fl*^; tumours. Right column: IF against pERK1/2shows regions rich in MAPK/ERK pathway activation in *Hesx1*^*Cre/*+^; *Ctnnb1*^*lox(ex3)/*+^ tumours. In contrast, *Hesx1*^*Cre/*+^; *Ctnnb1*^*lox(ex3)/*+^; *Trp53*^*fl/*+^ and *Hesx1*^*Cre/*+^; *Ctnnb1*^*lox(ex3)/*+^; *Trp53*^*fl/fl*^; tumours only showed scarce pERK1/2 positivity. **F** Plot of the proliferative index (percentage of KI67 + ve cells) in mouse tumour tissue sections, showing a significant increase in *Hesx1*^*Cre/*+^; *Ctnnb1*^*lox(ex3)/*+^; *Trp53*^*fl/fl*^ (n = 3, shown in red) in comparison to *Hesx1*^*Cre/*+^; *Ctnnb1*^*lox(ex3)/*+^; *Trp53*^*fl/*+^ (n = 4, shown in blue) tumours (*P* = 0.0241, unpaired t test). Horizonal lines represent the mean and standard deviation. **G** Plot of the pERK1/2 positivity index in mouse tumour sections, showing that despite a trend in increased pERK1/2 + cells in *Hesx1*^*Cre/*+^; *Ctnnb1*^*lox(ex3)/*+^; *Trp53*^*fl/fl*^ and *Hesx1*^*Cre/*+^; *Ctnnb1*^*lox(ex3)/*+^; *Trp53*^*fl/*+^ tumours, differences are not statistically significant (*p* = 0.2339, one-way ANOVA with Dunn’s multiple correction). Scale bars: b: 2.5 mm; d: 500 µm for top row and 100 µm for bottom row; e: 100 µm for left and middle columns, 200 µm for right column

To test the functional role of the *TP53* mutation identified in the malignant recurrent tumour, we generated *Hesx1*^*Cre/*+^; *Ctnnb1*^*lox(ex3)/*+^; *Trp53*^+*/*+^*, Hesx1*^*Cre/*+^; *Ctnnb1*^*lox(ex3)/*+^; *Trp53*^*fl/*+^ and *Hesx1*^*Cre/*+^; *Ctnnb1*^*lox(ex3)/*+^; *Trp53*^*fl/fl*^ mice with either wild-type *Trp53*, heterozygous or homozygous deletion of *Trp53*, respectively. As expected, the activation of oncogenic β-catenin resulted in the development of tumours in the three genotypes [37]. Control mice not carrying the oncogenic β-catenin allele (*Hesx1*^*Cre/*+^; *Trp53*^*fl/fl*^) did not develop any tumours. Interestingly, while the median survival of *Hesx1*^*Cre/*+^; *Ctnnb1*^*lox(ex3)/*+^; *Trp53*^*fl/*+^ mice was 500 days, this was significantly reduced to 220 days in *Hesx1*^*Cre/*+^; *Ctnnb1*^*lox(ex3)/*+^; *Trp53*^*fl/fl*^ mice (Fig. 4A). *Hesx1*^*Cre/*+^; *Ctnnb1*^*lox(ex3)/*+^; *Trp53*^+*/*+^ and *Hesx1*^*Cre/*+^; *Ctnnb1*^*lox(ex3)/*+^; *Trp53*^*fl/*+^ tumours were similar in size and histology (Fig. 4B). In contrast, *Hesx1*^*Cre/*+^; *Ctnnb1*^*lox(ex3)/*+^; *Trp53*^*fl/fl*^ tumours were significantly larger (Fig. 4B, C) and histologically, showed an increase of mitotic and necrotic bodies, whereas both features were rarely observed in *Hesx1*^*Cre/*+^; *Ctnnb1*^*lox(ex3)/*+^; *Trp53*^+*/*+^ and *Hesx1*^*Cre/*+^; *Ctnnb1*^*lox(ex3)/*+^; *Trp53*^*fl/*+^ tumours (Fig. 4D).

We then conducted a molecular analysis of the tumours by immunostaining, which showed a complete absence of p53 protein in *Hesx1*^*Cre/*+^; *Ctnnb1*^*lox(ex3)/*+^; *Trp53*^*fl/fl*^ tumours (Fig. 4E). Additionally, the number of Ki67-positive cells was significantly increased in *Hesx1*^*Cre/*+^; *Ctnnb1*^*lox(ex3)/*+^; *Trp53*^*fl/fl*^ in comparison with *Hesx1*^*Cre/*+^; *Ctnnb1*^*lox(ex3)/*+^; *Trp53*^*fl/*+^ tumours (unpaired t test, *P* = 0.0241) (Fig. 4F). Quantification of p-ERK1/2-positive cells showed a trend of decreased positivity in both *Hesx1*^*Cre/*+^; *Ctnnb1*^*lox(ex3)/*+^; *Trp53*^*fl/fl*^ and *Hesx1*^*Cre/*+^; *Ctnnb1*^*lox(ex3)/*+^; *Trp53*^*fl/*+^ tumours, although it was not significant when compared to *Hesx1*^*Cre/*+^; *Ctnnb1*^*lox(ex3)/*+^; *Trp53*^+*/*+^ (*p* = 0.2339) (Fig. 4E, G). Together, these data support the notion that loss of p53 expression results in the development of aggressive ACP tumours.

### Expression and methylation analyses identify differences in the tumour immune microenvironment between ACP and PCP

**Fig. 5:**
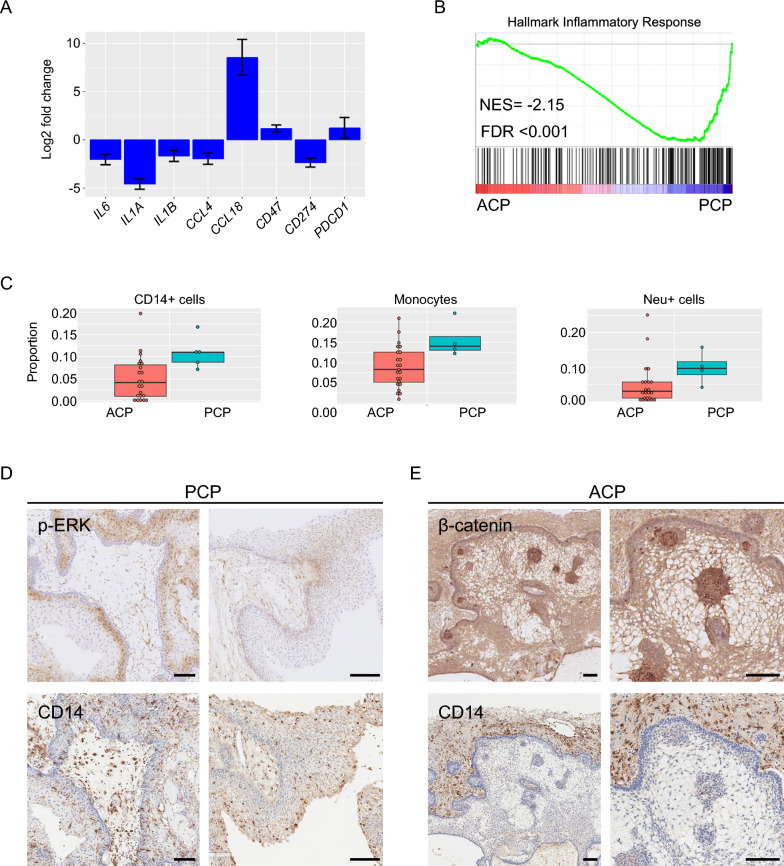
Molecular profiling identifies differences in the inflammatory infiltrates between ACP and PCP. **A** Differential expression of cytokines and immune checkpoint proteins between ACP and PCP. Positive fold change indicates upregulated in ACP relative to PCP. **B** GSEA plot showing enrichment of Hallmark Inflammatory Response geneset in PCP (NES = Normalised enrichment score; FDR = False Discovery Rate). **C** Plots showing distribution of CD14-positive, monocyte and neutrophil infiltrates in methylation patterns from ACP and PCP, as assessed by methylcibersort. **D**, **E** Immune infiltrate across tumour and reactive tissue as assessed by immunohistochemistry against CD14 in PCP (**D**) and ACP **E**. pERK1/2 and β-catenin staining are also shown demonstrating CD14 + ve cells throughout the epithelia and reactive glia of PCP, but limited the reactive glia in ACP. Scale bar: 100 µm

Differential expression and differential methylation analyses of primary versus recurrent ACP or PCP tumours failed to reveal any biologically relevant differences, likely suggesting a stable transcriptome and methylome during recurrence (Additional file 1 and Additional Table 4). In contrast, these analyses uncovered a significant upregulation of inflammation-related genes in PCP relative to ACP (Fig. 5A, B) (Further description in Supplementary Material). These findings were unexpected as ACP but not PCP has previously been associated with increased inflammation [22, 24].

To explore the immune infiltrate in the tumour types, we carried out deconvolution analysis on the methylation and expression datasets using MethylCibersort and Cibersort, respectively. Analysis of methylation profiles using signatures of immune cells derived from blood samples [47], identified a higher absolute proportion of CD14-positive cells within PCP compared with ACP (*p* = 0.016) (Fig. 5C, Additional Table 4). CD14 is expressed by macrophages and microglia, and to a lesser extent neutrophils and dendritic cells [48–50]. In agreement, using methylation signatures applied to immune infiltrates in brain tumours [51], we revealed a higher proportion of monocytes (*p* = 0.03) and neutrophils (*p* = 0.04) in PCP compared with ACP (Fig. 5C).

Analysis of gene expression profiles using Cibersort also revealed a trend towards both higher levels and increased activation of myeloid cells in PCP compared with ACP (Additional Fig. 4). Consistently, myeloid-related ontologies were also enriched in PCP (e.g., GO:0042119: neutrophil activation; GO:0002274: myeloid leukocyte activation). CD14 was upregulated in PCP compared to ACP (2.55 fold, *p* < 0.05) and notably, the transmembrane protein CD47, an inhibitor of myeloid cells known to be expressed in ACP, was upregulated in ACP relative to PCP (2.3 fold, adjusted *p*-value = 0.01) (Fig. 5A). Further analysis of the myeloid compartment in ACP using the single cell RNA sequencing dataset recently provided by Prince et al. [52] revealed a wide phenotypic and pathway activity diversity within macrophages/microglia in ACP (Additional file 1 and Additional Fig. 5).

To validate the results obtained from the computational analysis, we analysed the expression of CD14 in ACP and PCP histological sections by immunohistochemistry. There were CD14-positive cells in both ACP and PCP (Fig. 5D, E). However, whilst CD14-positive cells were detectable throughout the tumour epithelium in PCP, they were mostly observed within the reactive glia tissue, and were noticeably absent from the tumour epithelial, suggesting a specific local immune myelosuppressive phenotype in ACP (Fig. 5D, E). These data, together with the previous literature [22, 24, 30], demonstrate the presence of inflammatory infiltrates within both ACP and PCP and reveal distinct patterns and spatial relationships of immune cells in the two tumour types, with a remarkable lack of myeloid cell infiltration within the tumour epithelia in ACP.

## Discussion

Molecular profiling experiments of in vitro and in vivo models has advanced our understanding of the biology of craniopharyngioma, identified potential targetable pathways and led to several clinical trials evaluating novel therapies [4]. However, our understanding of the biological processes is limited and whether these and/or other oncogenic pathways are activated at recurrence/relapse has not been explored.

The results presented here demonstrate the clonal evolution within a subset of cases at recurrence, as evidenced by the acquisition of somatic CNVs. The mechanism of this clonal evolution is unclear, but cannot be wholly ascribed to radiotherapy-induced changes, as radiotherapy had only been administered in a subset of these cases. Analysis of WGS from a larger open access cohort of cases has confirmed the presence of such CNVs in a subset of ACP cases, including at primary resection. Although these results may suggest a more complex genomic landscape of ACP than the presence of *CTNNB1* alone, in the absence of conserved CNVs across the cohort, it is difficult to interpret the functional relevance of these genomic changes. Detailed analysis of WGS in clinically annotated longitudinally sampled ACP cohorts is likely to provide further understanding of the genomic landscape of ACP and its biological and clinical significance.

In contrast to the genomic landscape, in the subset of cases where data were available, methylation and transcriptional profiles in ACP remained stable during recurrence. This statement has limitations since the group of tumours analysed did not include some cases with aggressive phenotypes, including the malignant case, which showed large CNVs that may have resulted in differences in their transcriptome and methylome. However, when taking into consideration that these CNVs may be heterozygous and that the immunostaining against β-catenin and pERK1/2 remain unchanged in all primary/recurrent ACP cases analysed (except for the malignant relapsed tumour), the data suggest that certain pathways may be stable across recurrence in the majority of ACP tumours.

Understanding the pattern of gene pathway activation in the recurrence setting is important when considering novel therapies. Such approaches are often first used in the recurrence setting and therefore characterisation in this disease state is required. Importantly, in this study, we show that except for one malignant case, activation of the MAPK pathway is observed in all cases at recurrence regardless of their molecular features. Combined with previous preclinical testing supporting a class effect of MEKi in preclinical models and observations of correlation between pERK1/2 staining and Ki67 expression [22], these data support the basis of the CONNECT 2108 trial (NCT05286788) evaluating Binemetinib in recurrent craniopharyngioma and the use of MAPK inhibitor Tovarafanib (DAY101) T in the PNOC029 trial (NCT05465174). Importantly, however, this study highlights the heterogeneity of activation across and within individual tumours. Understanding the mechanisms driving MAPK activation and the possible correlations between clinical responses and patterns of activation are likely to be important in unmasking the role of these therapies in ACP patients. Of note, heterogeneity of MAPK pathway activation has also been observed in PCP, where pERK1/2 expression is detected in only a subset of cells, yet remarkable responses are achieved to targeted therapy [14–20].

Despite its designation as a “benign” tumour, a subset of craniopharyngiomas can behave very aggressively, some showing frequent recurrences despite optimal surgical and radiotherapy management. Understanding the biological process driving such behaviour is likely to be crucial for improving the outcomes of these patients. Analysis of the recurrent sample ACP11 highlights that loss of *TP53* may contribute to this aggressive behaviour in rare cases of ACP. Supporting this conclusion, the loss of p53 in a murine model of ACP results in fast-growing, malignant and aggressive tumours. In both mouse and human malignant tumours, MAPK pathway activation is significantly reduced or completely lost in the majority of tumour cells. This is clinically relevant since novel therapies (including off-the-shelf use of MAPK pathway inhibitors against ACP) are often first tested on aggressive cases, for which there are no therapeutic alternatives, and so it is recognised that responses or lack or response to these therapeutics may not be representative of their effects on less aggressive, more typical tumours. Testing novel therapies requires the design of clinical trials with matched biological studies to understand better the mechanisms of response.

Whilst the presence of the immune infiltrate within ACP has been previously characterised, the comparison between ACP and PCP has highlighted surprising differences that were somehow unexpected. In particular, the distribution of macrophage/microglia in PCP and ACP strongly suggests the presence of an immune myelosuppressive environment in ACP. CD14-positive cells are found only within the glial reactive tissue in ACP, whilst they are distributed throughout the tumour epithelium in PCP. The absence of CD14-positive cells within the tumour epithelium in ACP is unexpected as the β-catenin-accumulating epithelial whorls (cell clusters) express a variety of chemo-attractant cytokines (e.g., members of the CCL and CXCL family of chemokines including CCL2, CXCL1, CXCL3, CXCL11) [22, 36, 38]. Local immunosuppressive signalling, e.g., via CD47 may contribute to this exclusion and this signalling has also been suggested as contributing to MAPK pathway activation within the palisading epithelia [21].

## Conclusions

The data presented here have provided a better understanding of relapsed craniopharyngioma by revealing clonal evolution in a subset of tumours, uncovering a relatively stable transcriptome and methylome during recurrence in ACP, including the activation of the MAPK pathway in the vast majority of relapsed tumours, and identifying an ACP case with clear malignant progression at recurrence. Additionally, we have revealed the presence of an immune infiltrate that is consistent with an immune myeloid suppressive environment in ACP but not in PCP, despite the well-recognised relevance of inflammation in ACP. Together, these data support the use of MAPK pathway inhibitors and immunomodulatory therapies against ACP.

### Supplementary Information

Additional file 1.

Additional file 2.

Additional file 3.

Additional file 4.

Additional file 5.

Additional file 6.

Additional file 7.

Additional file 8.

Additional file 9.

Additional file 10.

## Data Availability

Results of analyses are presented in Additional Tables. Due to governance restrictions, we are unable to deposit raw data of methylation or sequencing files, however the authors may be contacted if there are specific queries.
