# Supplementary material for: Recurrent adamantinomatous craniopharyngiomas show MAPK pathway activation, clonal evolution and rare TP53-loss-mediated malignant progression
Source: Acta Neuropathol Commun. 2024 Aug 10;12:127. doi: 10.1186/s40478-024-01838-4 (PMC11316312; doi:10.1186/s40478-024-01838-4)
Supplement: Supplementary file 1 — Additional file 1. [file 40478_2024_1838_MOESM1_ESM.docx]

**Additional Material**

**Additional Materials and Methods**

**Bioinformatic analyses**

*Methylation array analysis*: For differential methylation analysis data were normalised according to the preprocessIllumina implementation within minfi (v.1.28.4), using Bioconductor in R version 3.5.3^1^. Probes located on the X and Y chromosomes were removed and those with missing values in any sample were omitted across the cohort. Differential methylation probe analysis was performed using Limma with Benjamini-Hochberg correction and differential methylation region analysis using DMRcate with lambda=1000, C=2^2^. Samples were analysed withversion v11b6 of the DKFZ Molecular Neuropathology (MNP) brain tumour classifier (“Heidelberg Classifier”) ^3^.

Copy-number changes for the cohort were identified from EPIC arrays using R version 4.2.1 and the R package conumee (v 1.30.0), as previously described^4^. GISTIC2.0^5^ was applied to binned copy number data to identify recurrent changes, using log2 ratio cutoffs of 0.12 and -0.15 respectively to denote chromosomal gain/loss. These cutoffs were empirically determined from confirmed chromosomal changes. Annotation is with hg38 genome coordinates.

*RNA Seq analysis*: Sequencing reads were aligned using samtools version 1.8 (Li et al., 2009) and Picard tools version 2.21.8 to the hg38 reference sequence and Ensembl transcripts (build 99) using STAR v2.5.3 ^6^. Aligned reads were filtered for unique mapping reads, and putative PCR duplicates were removed. Read summarization was done using featureCounts^7^.

Exon 3 *CTNNB1* mutations were confirmed using Integrative Genomics Viewer. Differential expression was performed using DESeq2 (v.1.22.2) as previously described ^8^, with paired analysis of primary and recurrent tumours as appropriate (summarised in Results/Additional Results).

For Cibersort analysis of RNASeq data, Transcripts Per Million (TPM) normalised expression data were used. Data were uploaded to the CIBERSORTx portal (https://cibersortx.stanford.edu/) and analysed in absolute mode with 1000 permutations. B-mode batch correction was used for RNA. Wilcoxon rank test was used to compare cell infiltrates between ACP and PCP. For methylation data, before Cibersort analysis, data were prepared using the Prep.CancerType function of MethylCibersort, using the peripheral immune cell reference signatures ^9^ and the glioma signature matrix generated by Grabovska *et al.,*^10^. Mann-Whitney U test was used to compare for differences between tumour types.

*Analysis of Copy numbers in WGS data:* Chromosomal arm-level changes were scored from segmented copy number data as previously described^11^. Whilst the CBTN online repository reports many smaller focal structural variants, the vast majority of these are peri-centromeric, and/or are in regions of population structural variation and therefore difficult to interpret whether genuinely somatically acquired. Further validation, beyond the scope of this paper, would be required to validate these variants. We therefore sought to assess the prevalence of larger scale (i.e.*,* chromosome-arm level) alterations, to compare against chromosomal changes observed in the methylation array cohort.

*Analysis of single cell RNA sequencing Data* The basis for the initial data utilized in this study stemmed from previously acquired single-cell and single-nucleus RNA sequencing data (sc/snRNA-seq)^12^. Our custom analysis used the R statistical programming language (v4.2). We employed several publicly available libraries within R to perform extensive statistical analyses. The Seurat library was used for pre-processing, scaling, clustering, and dimension reduction^13^. It enabled the calculation of differentially expressed genes, identifying critical elements in our datasets. We utilized ggplot2 to create appealing bar charts and boxplots for our data analysis ^14^. The theming and exporting capabilities of ggplot2 were also used to generate high-quality, vectorized files suitable for publication. The escape package was also employed to run the UCell enrichment program on the M1/M2 genes^15,16^. This package helped us interpret cellular heterogeneity and the interplay of the gene sets at a single-cell resolution. Metascape was used to determine ontology enrichment among differentially expressed genes^17^. The M1/M2 gene sets originated from^18^. This comprehensive toolset facilitated annotation and interpretation of data, providing a more holistic understanding of our findings.

**Antibodies and epitope retrieval for IHC and immunofluorescence**

**Immunohistochemistry**

***Human:***

pERK1/2 (197G2)– 1:50, H1 20 mins epitope retrieval

B-Cat (clone Bcat-1 code M3539) -1:100, H1 20 mins epitope retrieval

P21 (Waf1/Cip1, 12D1)-1/50 H2 10 mins epitope retrieval

TP53 (Clone DO-7)- 1/50 H1 20 mins epitope retrieval

CD14 (Clone 3653) -Ready made/no dilution, H1 20 mins epitope retrieval

***Murine:***

TRP53 (clone CM5) – 1:100 Tris-EDTA pH 9, 20 min epitope retrieval

**Immunofluorescence**

| **Target** | **Species** | **Clonality** | **Clone** | **Company** | **Catalog Number** | **Dilution** | **Retrieval Method** | **Amplification** |
| --- | --- | --- | --- | --- | --- | --- | --- | --- |
| **Ki67** | Rabbit | Monoclonal | SP6 | Abcam | ab16667 | 1:100 | Tris-EDTA pH9.0 | Y |
| **p53** | Rabbit | Polyclonal | CM5 | Leica | NCL-p53-CM5p | 1:100 | Tris-EDTA pH9.0 | Y |
| **β-Catenin** | Mouse | Monoclonal | 6F9 | Sigma | C7082 | 1:300 | Tris-EDTA pH9.0 | N |
| **Cleaved Caspase 3** | Rabbit | Polyclonal | Asp175 | Cell Signaling | 9661 | 1:300 | Tris-EDTA pH9.0 | Y |
| **p-p44/42 MAPK (Erk 1/2)** | Rabbit | Monoclonal | 197G2 | Cell Signaling | 9101/  4377S | 1:1000 | Tris-EDTA pH9.0 | N |

**Phenotyping of murine tumours**

Tumours were imaged in brightfield under a stereomicroscope (Leica) using 0.8X magnification and a constant exposure of 3.7 seconds. Samples to be used for immunohistochemistry were washed in phosphate-buffered saline (PBS) and immediately placed in ice-cold freshly made 4% paraformaldehyde (PFA), fixed over-night and posteriorly dehydrated using ethanol (EtOH) gradients. Dehydrated samples were then paraffin-embedded and cut into 6 μm-thick sections using a rotary microtome (Leica) and mounted on Superfrost Plus slides (Thermo) in series to have a representation of the whole tumour per analysed slide. When possible, all experiments performed on mouse tumours were performed with a minimum of three biological replicates from at least two different litters.

For measurement of mean tumour size, freshly dissected tumours were imaged at 0.8X magnification in a Leica MZ FLIII stereomicroscope connected to a Leica DC500 camera. The scale of the image was then calculated from graticule images at the same magnification and Fiji/ImageJ^19^ software was used to measure tumour sizes. Tumour diameters were calculated by averaging the longest side of the tumour and its perpendicular. Immunostaining for Ki67 and pERK1/2 was conducted following a previously described protocol^20^. Information regarding antibody concentrations and antigen retrieval conditions can be found in the supplementary material. Quantification of positive cells in immunofluorescence images was conducted in QuPath ^21^ using the positive cell detection function with the following parameters: Detection channel: DAPI, requested pixel size: 0.1 µm, background radius: 20 µm, median filter radius: 1 µm, Sigma: 1.5 µm, minimum area: 10 µm^2^, maximum area: 200 µm^2^, threshold 1000 µm, cell expansion: 0 µm, score compartment: AF488 mean, threshold 1: 9197, threshold 2: 8313, threshold 3: 7430.

**Additional Results**

**Further description of ACP cases with copy number variations**

Three of the cases with acquired copy number of changes in recurrence samples were known to have received radiotherapy between primary resection and recurrence (ACP1,9,11). In two cases (ACP6,10), no radiotherapy had been administered, suggesting that CNVs cannot be fully explained by the effects of radiation (**Additional Tables 1 and 2**). Whether ACP2 was irradiated was unknown. Four of the cases with the acquisition of CNVs (ACP1,9,10,11) exhibited particularly aggressive behaviour including needing multiple subsequent resections and/or showing malignant histology (outlined in **Additional** **Tables 1 and 2**).

Detailed CNV analysis with GISTIC confirmed recurrent losses (0.5 - 28.5Mb) across cases of overlapping areas of chromosome 3 (Chr3: 42258509 - 70800849) (ACP 9, 11), chromosome 5 (chr5: 140,620,416 - 141,070,415; chr5: 146,570,438 - 147,270,437; chr5: 153,770,441 - 171,272,996) (ACP9,10) and chromosome 15 (Chr 15: 29657797 – 55557802) (ACP2,9) across the cohort (**Figure 1 A**). No other CNVs were observed across the ACP cohort, and no CNVs at all were detected in the PCP tumours (n=7) (**Additional Figure 1**).

Although we could not determine that the same exon 3 *CTNNB1* mutation was present in the primary and recurrent tumours in all cases, due to insufficient data, in 6 of 11 cases we could demonstrate the presence of identical mutations across samples, confirming a clonal relationship between primary tumours and recurrences (Full details in **Additional Table 2**).

Of the recurrent changes identified in the methylation array cohort, chromosomal arm copy number loss of Chr 5 and 15 were also observed in the CBTN cohort in one and two samples, respectively (**Additional Figure 1B**).

**Gene expression analysis of craniopharyngioma**

Since RNAseq was carried out from FFPE tumour samples, some of which have been stored for many years at room temperature, we first analysed whether the RNAseq data was biologically relevant. As expected, clustering analysis of gene expression patterns revealed that ACP (n=15) and PCP (n=5) tumours clustered separately (**Additional Figure 3A**). Differential expression and ontology analyses of the 500 most upregulated genes in ACP relative to PCP showed the enrichment in ACP tumours for genes related to the WNT pathway (adjusted p-value <0.001), cell-cell signalling (adjusted p<-0.001) and tissue development, including odontogenesis (adjusted p-value <0.001) (**Additional Table 4**). These results are concordant with previous transcriptomic studies of ACP ^8,22,23^. Conversely, differential expression and ontology analyses of the 500 most upregulated genes in PCP relative to ACP confirmed activation of the MAPK pathway and epidermis development ontologies in PCP (p=0.002)) (**Additional Table 4**) as would be expected in this tumour type. Finally, we used gene set enrichment analysis (GSEA) to compare these FFPE-derived datasets with the 500 most upregulated genes in ACP or PCP that were identified using frozen tumour tissue in previously published studies ^22^. This analysis revealed significant enrichment scores between the FFPE- and frozen-gene expression datasets (ACP: NES= 3.76; PCP: NES =4.56; FDR<0.001 for both tumour comparisons) (**Additional Figure 3B**)^22^. These analyses also confirmed upregulation in ACP of a range of signalling factors known to be upregulated in ACP when compared to pituitary tissue and non-functioning pituitary adenoma, including *WNT7A* (5.25 fold, padj 1.21x10^-6^), *FGF4* (7.18 fold, padj=3.11x10^-7^), *BMP7* (3.4 fold, padj=1.39 x10^-8^), S*HH* (3.7 fold, padj=0.01), *EDA* (3.68 fold, padj 0.0004). Others, known to be upregulated in ACP, such *as FGF3, BMP4,* and *TGFβ* were not differentially expressed, suggestive of upregulation in both ACP and PCP compared to control tissues. Growth factors upregulated in PCP included *BMP2* (4.2 fold, padj =1.97x10^-7^), *WNT9A* (3.86 fold, padj= 2.7x10^-6^) **(Additional Figure 3C).** Together, these studies confirm that the sequencing of our archival FFPE tumours was successful and the data biologically meaningful.

Paired (i.e., comparing each of the primary tumours with their respective relapsed tumours as individual tumour pairs; n=8 samples) and unpaired (i.e., comparing all primary tumours with all relapsed samples; n=15 samples) analyses revealed that fewer than 25 genes were differentially expressed between primary and recurrent ACP, and none of these genes were associated with specific pathways or appeared biologically relevant, suggesting stochastic transcriptional changes.

Although we sought to analyse the expression profiles of ACP tumours with or without CNVs (e.g., ACP6, 9 and 11), unfortunately, there was insufficient RNA sequencing data to evaluate any differences.

**Further analysis of immune response differences between ACP and PCP**

Differential expression analysis between ACP and PCP uncovered a significant upregulation of inflammation-related genes in PCP relative to ACP, including cytokines (IL1A:23 fold; IL6:4 fold; IL1B:3 fold; CCL4:4 fold; adjusted p-value < 0.05) (**Figure 5A**). This was supported by gene ontology analysis, which also highlighted an immune cell activation (e.g., GO:00042119: neutrophil activation; GO:0002444: myeloid leukocyte-mediated activation) in PCP relative to ACP (**Additional Table 4C**). Moreover, GSEA revealed enrichment for inflammation-related hallmark gene sets in PCP compared with ACP (e.g., hallmark inflammatory response, NES=2.15, FDR<0.01) (**Figure 5B**). In contrast, expression of the anti-inflammatory cytokine CCL18 was upregulated in ACP relative to PCP (365 fold, adjusted p= 0.0001) (**Figure 5A**). Of relevance, the expression of glial markers, such as GFAP or OLIG2, was not differentially expressed between ACP and PCP, indicating that the differences in the expression of immune regulators were not due to the presence of unequal content of glial reactive tissue between the ACP and PCP tumour samples (**Additional Table 4A**).

Whilst Cibersort suggested a higher proportion of CD8 T cells in PCP (p=0.008), this was not significant in the methylation data (**Additional Figure 4**), and genes of lymphoid markers (e.g., *CD4, CD8A, CD8B*) were not significantly differentially expressed between ACP and PCP (**Additional Table 4**). CD274, which encodes PDL1 was upregulated in PCP compared with ACP (5 fold, adjusted p-value<0.01), whereas PD1 was not differentially expressed (**Figure 6a**). No consistent significant differences in immune cell infiltrate were identified between primary and recurrence tumours on analysis of MethylCibersort or Cibersort data (data not shown)**.**

**Molecular characterisation of the myeloid compartment in ACP**

To further characterise the phenotype of myeloid cells in ACP, we performed an in-depth analysis of macrophages and microglia, This confirmed the prominence of macrophages as the principal immune cell population within ACP tissue (**Additional Figure 5A)**. After analysing the ACP macrophage population, we identified 23 distinct gene expression states using mutual nearest neighbour clustering **(Figure Figure 5C)**. These groups included both M1 and M2 polarizations, among others (**Additional Figure 5C**). When the analysis was further restricted by cluster ID, most clusters were characterized by a higher M2 score than an M1 score (**Additional Figure 5D**). Exploring the biological pathways within each cluster, for M1 clusters, specifically clusters 11 and 22, the differentially expressed genes (n=57 and n=7, respectively) were found to be associated with multiple biological processes; these included peptide chain elongation, eukaryotic translation elongation and ribosomal processes. Moreover, they were implicated in antigen processing, the presentation of exogenous peptide antigens via MHC class II, neutrophil degranulation and leukocyte activation (**Additional Figure 5E**). Conversely, the differentially expressed genes for M2 clusters, specifically clusters 3 and 4 (n=238 and 159, respectively), were associated with cell activation, positive regulation of cytokine production, PI3K-Akt signalling pathway, matrix metalloproteinase regulation and p53 activation (**Additional Figure 5F**). Together, these data demonstrate a wide phenotypic and pathway activity diversity within macrophages/microglia in ACP.

**Additional Figure Legends**

**Additional Figure 1:** **Chromosomal alterations in tumour samples.** **A.** Heatmap showing copy number changes identified across whole cohort of craniopharyngioma samples. **B.** Chromosomal arm copy number changes identified in whole genome sequencing from seven cases from the Children’s Brain Tumour Network. Red indicates gain, blue indicates loss.

**Additional Figure 2: Heterogeneity of the pattern of pERK1/2 staining across tumours.** Scale bar = 500µm and 100µm for low and high power respectively. This shows that there is diversity of p-ERK expression within and across cases with isolated areas of positive staining within the tumour epithelium (e.g. samples 1.0, 1.1, 2.1,2.2). Other have more extensive staining at the tumour, reactive tissue boundary (e.g. samples 4.1, 4.2., 5.1.,5.2, 7.1, 12, 14) in both epithelium and glia, and others with predominant staining in the reactive glia (e.g 3.2). Staining may also be seen around epithelial whorls (e.g. 4.1, 12) and as per Figure 2.

**Additional Figure 3: Distinct molecular profiles in ACP and PCP.** **A.** Clustering of samples based on RNA-seq expression data showing separation of ACP and PCP. **B.** Gene set enrichment analysis confirming concordance with analysis of fresh tissue by *Holsken et al.* (22). Geneset made of top 500 genes up in ACP and top 500 up in PCP and GSEA run using ranked differential expression list from *Holsken et al.* (22) **C.** Differential expression of genes between ACP and PCP. Differential expression confirmed upregulation in ACP of a range of signalling factors known to be upregulated in ACP when compared to pituitary tissue and non-functioning pituitary adenoma, including *WNT7A* (5.25 fold, padj 1.21x10^-6), *FGF4* (7.18 fold, padj=3.11x10-7), *BMP7* (3.4 fold, padj=1.39 x10-8), S*HH* (3.7 fold, padj=0.01), *EDA*(3.68 fold, padj 0.0004). Others, known to be upregulated in ACP, such *as FGF3, BMP4, TGFβ* were not differentially expressed, suggestive of upregulation in both ACP and PCP compared to control tissues. Growth factors upregulated in PCP included *BMP2* (4.2 fold, padj =1.97X10-7) *WNT9A* (3.86 fold, padj= 2.7X10-6).

**Additional Figure 4: In silico deconvolution of methylation and RNAseq datasets.** **A, B.** Methylcibersort deconvolution of immune infiltrate in craniopharyngioma (red, ACP; green, PCP). **C.** Cibersort deconvolution of immune infiltrate derived from RNA sequencing. A and C used signatures derived from analysis of immune cells in blood samples (Chen *et al.,* 2018 and Chakrovarthy *et al,.* 2018) B used signatures derived from paediatric brain tumour samples (Grabovska *et al,.* 2020). Neu = Neutrophil, Treg = regulatory T cell, Eos = eosinophil, Mono = Monocyte.

**Additional Figure 5. A heterogenous macrophage/microglia population is prominent in ACP.**

1. Single-nuclei and single-cell dataset from Prince et al,. 2023, reveals that macrophages are the most prominent immune cell type in ACP. The UMAP and bar chart both highlight macrophages in red. **B**. The macrophage population is diverse, with 23 distinct clusters identified using unbiased optimization. Each color represents a unique cluster, as shown in the legend to the right. The UMAP plots display the M1 and M2 enrichment scores, with red and green points representing M1-high and M2-high cells and nuclei, respectively. Blending the M1 and M2 scores indicates that only a small number of these myeloid cells are in either an M1 or M2 states (indicated by yellow points), with the majority of these cells sharing features of both states. The legend on the right side of the plot shows the color scale for the blended plot, with grey points representing cells and nuclei that are low for both M1 and M2. **C.** The boxplots show the M1/M2 enrichment score ratio grouped by cluster ID. The red line at 1 represents when M1 and M2 scores are equal. Most clusters have a mean M1/M2 ratio below 1, indicating a mainly M2 signature (shown by the green arrow on the right side). However, a few clusters have values above 1, meaning a primarily M1 signature (indicated by the purple arrow on the right). The colored points on the X-axis match the cluster IDs in panel b. D. Ontology enrichment for M1-high clusters (clusters 11 and 22, left and right, respectively). **E.** Ontology enrichment for M2-high clusters (clusters 3 and 4, left and right, respectively), showing differences in pathways active within distinct macrophage populations.

**References for Additional Material**

**1.** Aryee MJ, Jaffe AE, Corrada-Bravo H, et al. Minfi: a flexible and comprehensive Bioconductor package for the analysis of Infinium DNA methylation microarrays. *Bioinformatics.* 2014; 30(10):1363-1369.

**2.** Peters TJ, Buckley MJ, Chen Y, Smyth GK, Goodnow CC, Clark SJ. Calling differentially methylated regions from whole genome bisulphite sequencing with DMRcate. *Nucleic Acids Res.* 2021; 49(19):e109.

**3.** Capper D, Jones DTW, Sill M, et al. DNA methylation-based classification of central nervous system tumours. *Nature.* 2018; 555(7697):469-474.

**4.** Sharma T, Schwalbe EC, Williamson D, et al. Second-generation molecular subgrouping of medulloblastoma: an international meta-analysis of Group 3 and Group 4 subtypes. *Acta Neuropathol.* 2019; 138(2):309-326.

**5.** Mermel CH, Schumacher SE, Hill B, Meyerson ML, Beroukhim R, Getz G. GISTIC2.0 facilitates sensitive and confident localization of the targets of focal somatic copy-number alteration in human cancers. *Genome Biol.* 2011; 12(4):R41.

**6.** Dobin A, Davis CA, Schlesinger F, et al. STAR: ultrafast universal RNA-seq aligner. *Bioinformatics.* 2013; 29(1):15-21.

**7.** Liao Y, Smyth GK, Shi W. featureCounts: an efficient general purpose program for assigning sequence reads to genomic features. *Bioinformatics.* 2014; 30(7):923-930.

**8.** Apps JR, Carreno G, Gonzalez-Meljem JM, et al. Tumour compartment transcriptomics demonstrates the activation of inflammatory and odontogenic programmes in human adamantinomatous craniopharyngioma and identifies the MAPK/ERK pathway as a novel therapeutic target. *Acta Neuropathol.* 2018; 135(5):757-777.

**9.** Chakravarthy A, Furness A, Joshi K, et al. Pan-cancer deconvolution of tumour composition using DNA methylation. *Nat Commun.* 2018; 9(1):3220.

**10.** Grabovska Y, Mackay A, O'Hare P, et al. Pediatric pan-central nervous system tumor analysis of immune-cell infiltration identifies correlates of antitumor immunity. *Nat Commun.* 2020; 11(1):4324.

**11.** Goschzik T, Schwalbe EC, Hicks D, et al. Prognostic effect of whole chromosomal aberration signatures in standard-risk, non-WNT/non-SHH medulloblastoma: a retrospective, molecular analysis of the HIT-SIOP PNET 4 trial. *Lancet Oncol.* 2018; 19(12):1602-1616.

**12.** Prince E, Apps J, Jeang J, et al. Unraveling the Complexity of the Senescence-Associated Secretory Phenotype in Adamantinomatous Craniopharyngioma using Multi-Modal Machine Learning Analysis. *Neuro-Oncology.* Under Revision November 2023.

**13.** Hao Y, Hao S, Andersen-Nissen E, et al. Integrated analysis of multimodal single-cell data. *Cell.* 2021; 184(13):3573-3587 e3529.

**14.** Wickham H. *ggplot2: Elegant Graphics for Data Analysis*: Springer-Verlag New York; 2016.

**15.** Borcherding N, Vishwakarma A, Voigt AP, et al. Mapping the immune environment in clear cell renal carcinoma by single-cell genomics. *Commun Biol.* 2021; 4(1):122.

**16.** Andreatta M, Carmona SJ. UCell: Robust and scalable single-cell gene signature scoring. *Comput Struct Biotechnol J.* 2021; 19:3796-3798.

**17.** Zhou Y, Zhou B, Pache L, et al. Metascape provides a biologist-oriented resource for the analysis of systems-level datasets. *Nat Commun.* 2019; 10(1):1523.

**18.** Jiang Y, Yang J, Liang R, et al. Single-cell RNA sequencing highlights intratumor heterogeneity and intercellular network featured in adamantinomatous craniopharyngioma. *Sci Adv.* 2023; 9(15):eadc8933.

**19.** Schindelin J, Arganda-Carreras I, Frise E, et al. Fiji: an open-source platform for biological-image analysis. *Nat Methods.* 2012; 9(7):676-682.

**20.** Gonzalez-Meljem JM, Ivins S, Andoniadou CL, Le Tissier P, Scambler P, Martinez-Barbera JP. An expression and function analysis of the CXCR4/SDF-1 signalling axis during pituitary gland development. *PLoS One.* 2023; 18(2):e0280001.

**21.** Bankhead P, Loughrey MB, Fernandez JA, et al. QuPath: Open source software for digital pathology image analysis. *Sci Rep.* 2017; 7(1):16878.

**22.** Holsken A, Sill M, Merkle J, et al. Adamantinomatous and papillary craniopharyngiomas are characterized by distinct epigenomic as well as mutational and transcriptomic profiles. *Acta Neuropathol Commun.* 2016; 4:20.

**23.** Gump JM, Donson AM, Birks DK, et al. Identification of targets for rational pharmacological therapy in childhood craniopharyngioma. *Acta Neuropathol Commun.* 2015; 3:30.
