# Supplementary figures and images for: Recurrent adamantinomatous craniopharyngiomas show MAPK pathway activation, clonal evolution and rare TP53-loss-mediated malignant progression
Source: Acta Neuropathol Commun. 2024 Aug 10;12:127. doi: 10.1186/s40478-024-01838-4 (PMC11316312; doi:10.1186/s40478-024-01838-4)

A

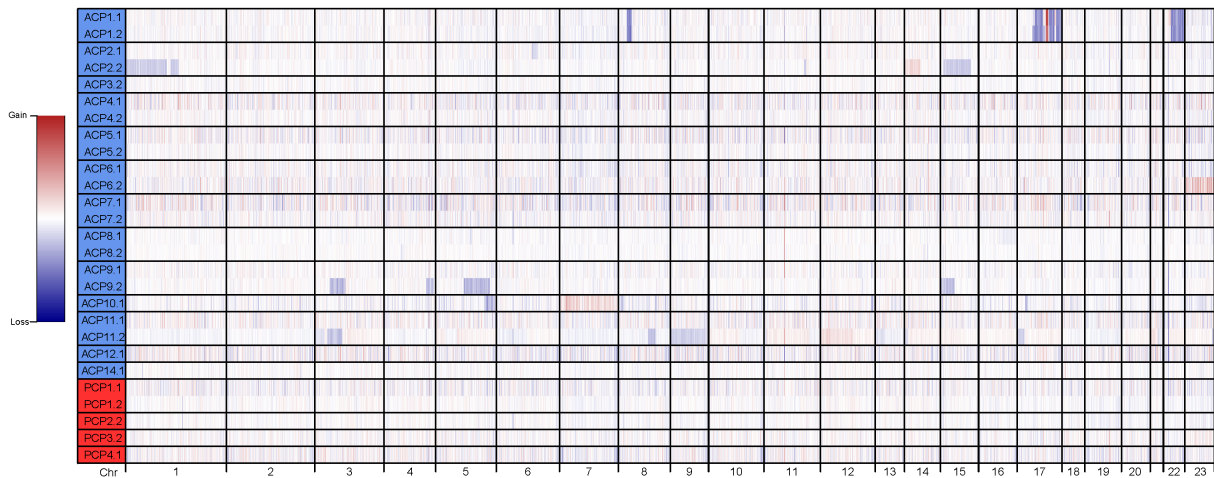

B

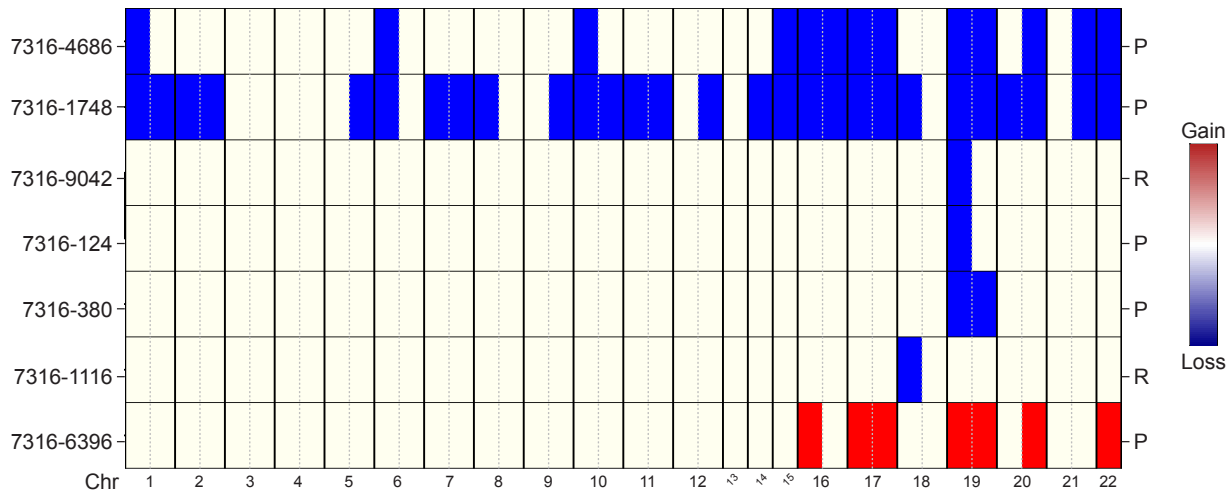

Supplement: Supplementary file 2 — Additional file 2. [file 40478_2024_1838_MOESM2_ESM.pdf]

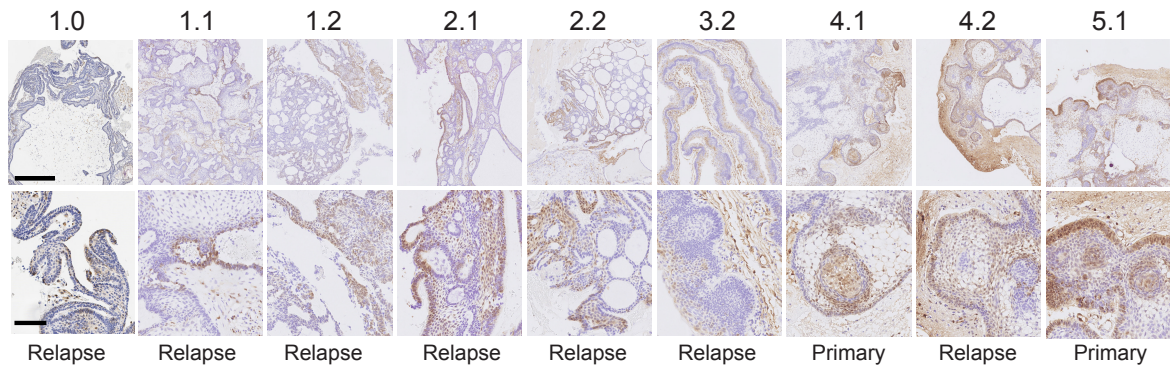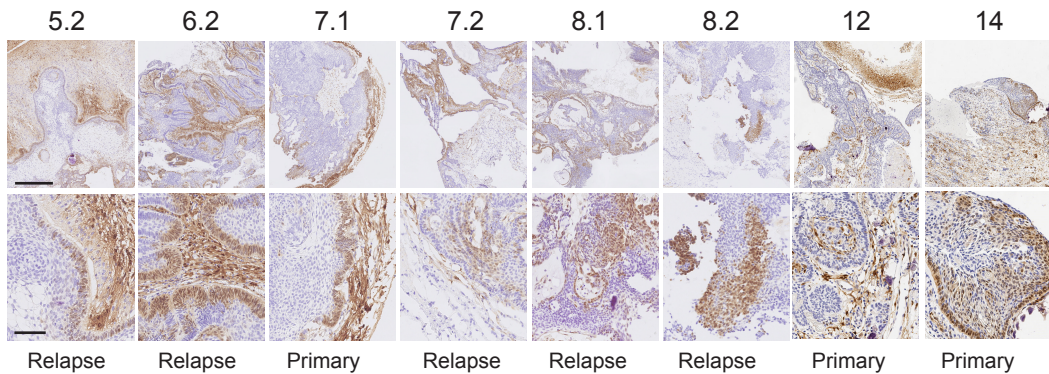

Supplement: Supplementary file 3 — Additional file 3. [file 40478_2024_1838_MOESM3_ESM.pdf]

A

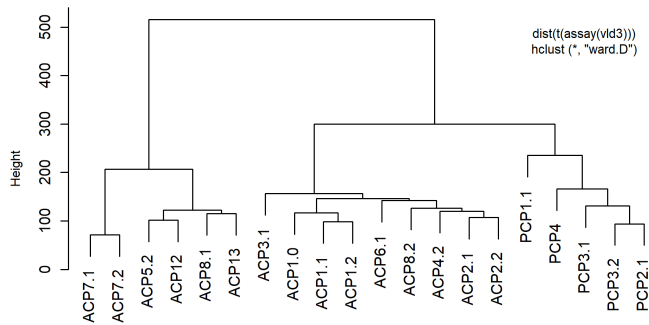

B

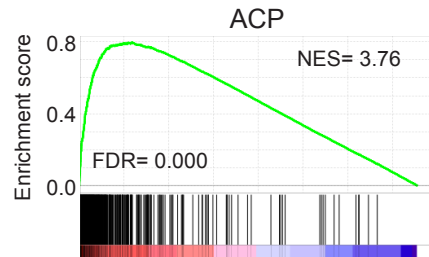

C

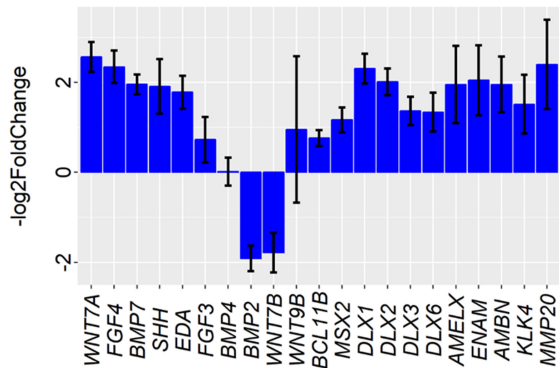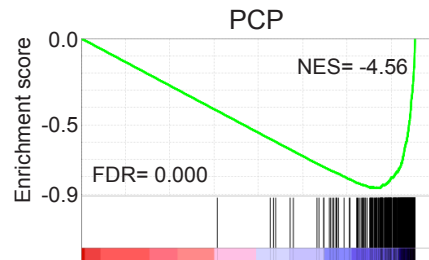

Supplement: Supplementary file 4 — Additional file 4. [file 40478_2024_1838_MOESM4_ESM.pdf]

a

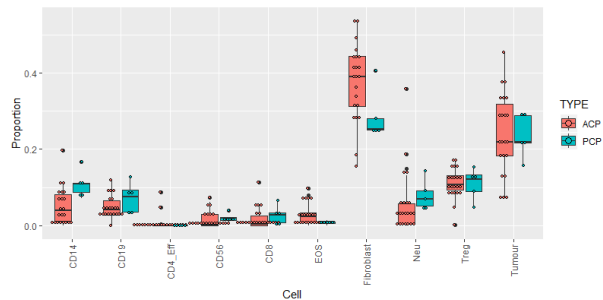

b

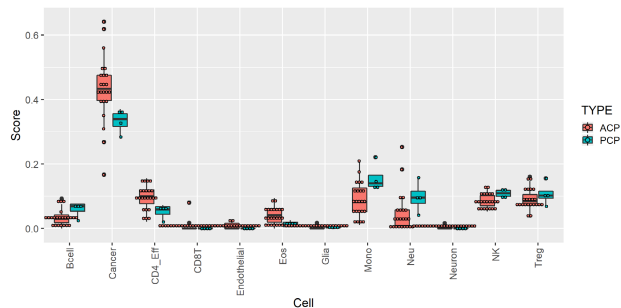

c

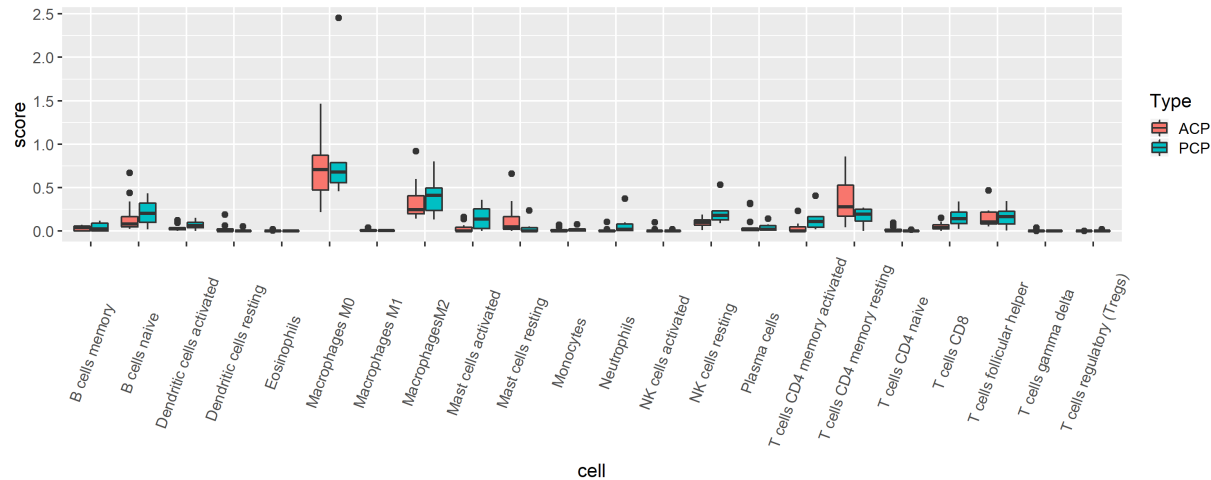

Supplement: Supplementary file 5 — Additional file 5. [file 40478_2024_1838_MOESM5_ESM.pdf]

A

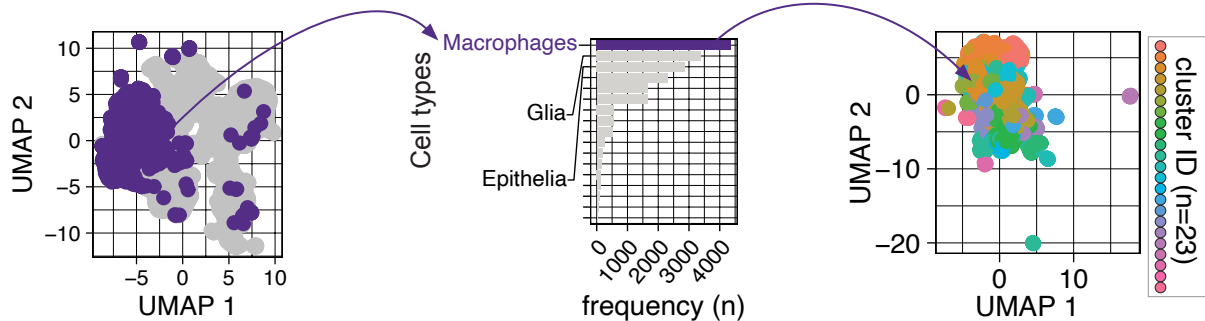

B

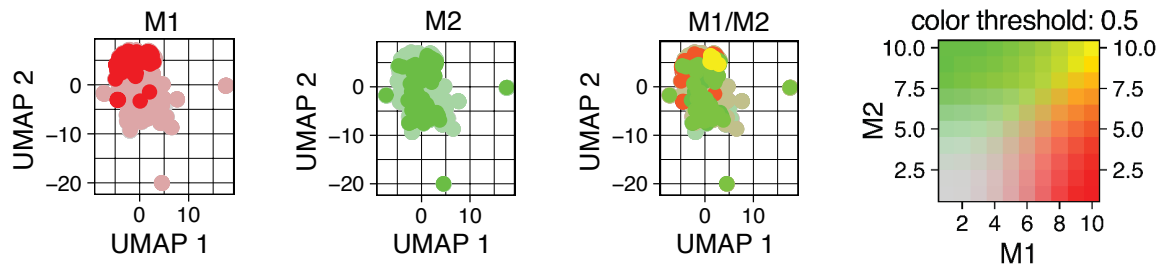

C

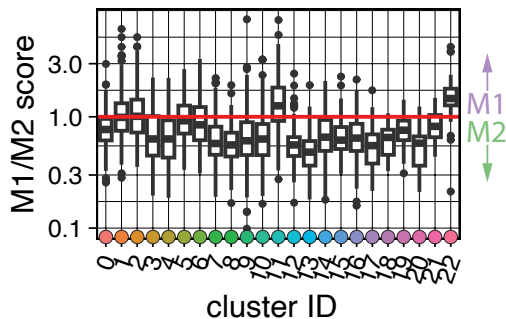

D

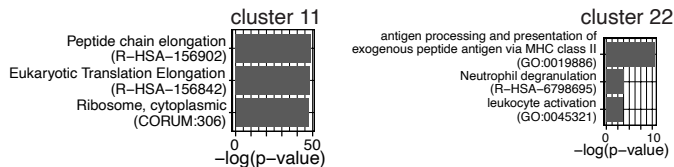

E

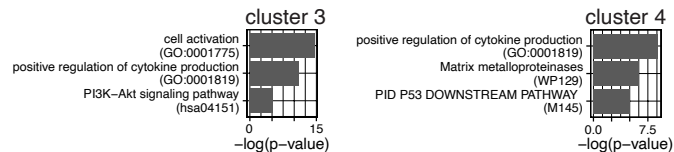

Supplement: Supplementary file 6 — Additional file 6. [file 40478_2024_1838_MOESM6_ESM.pdf]
