# Supplementary material for: Recurrent adamantinomatous craniopharyngiomas show MAPK pathway activation, clonal evolution and rare TP53-loss-mediated malignant progression
Source: Acta Neuropathol Commun. 2024 Aug 10;12:127. doi: 10.1186/s40478-024-01838-4 (PMC11316312; doi:10.1186/s40478-024-01838-4)
Supplement: Supplementary file 7 — Additional file 7. [file 40478_2024_1838_MOESM7_ESM.docx]

**Additional Table 1: Clinical information and *CTNNB1* mutations in tumours used in this study.**

| **Case** | **Age at diagnosis** | **Case summary** | **Samples analysed** | **Mutations** |
| --- | --- | --- | --- | --- |
| **Recurrent ACP cases** | | |  |  |
| ACP1 | Paediatric | Aggressive relapsing/progressing case, requiring frequent (>20) resections. Received 50Gy in 30 fractions 5 years after diagnosis and Intra-cystic ^32^P 11 years after diagnosis | 3 x recurrences, 5, 14, and 24 years post-diagnosis | *CTNNB1* S33P |
| ACP2 | Adult | Recurrences 14 and 16 years following initial diagnosis. Not known if received radiotherapy | 2 x recurrences | *CTNNB1* D32N |
| ACP3 | Adult | Relapse 5 years after initial resection, no radiotherapy | 1 x primary 1 x recurrence | *CTNNB1* S37W |
| ACP4 | Paediatric | Recurrences 1 and 7 years after initial cystic presentation and radiotherapy | 2 x recurrence | *CTNNB1* S37F |
| ACP5 | Adult | Recurrence 20 years after initial gross total resection. No prior RT | 1 x primary 1 x recurrence | *CTNNB1* D32Y |
| ACP6 | Adult | Recurrence 2 years after initial gross total resection. No prior RT | 1 x primary 1 x recurrence | NA |
| ACP7 | Adult | Recurrence 7 years after initial gross total resection. No prior RT | 2 x recurrences | *CTNNB1* D32G |
| ACP8 | Paediatric | Recurrences 14 and 21 years after initial presentation | 2 x recurrences | *CTNNB1* T41I |
| ACP9 | Paediatric | Initial gross total resection. Recurrence from within 1 year of initial surgery, treated with radiotherapy.  Further multiple & multifocal recurrences, including at distant sites. Treated with multiple surgical resections, stereotactic radiosurgery, trial of systemic pegylated IFN. Intracystic IFN for cystic recurrences. No malignant features histologically | 1 x primary 1 x recurrence following described treatments | *CTNNB1* S37F |
| **ACP with malignant features at recurrence** | |  |  |  |
| ACP10 | Paediatric | Initial resection only. No radiotherapy. Recurrence 17 years later with malignant histology (i.e. poorly differentiated epithelial tumour with high mitoticcount - 30 per 10 high power field). Diffusely infiltrative. | 1 x recurrence | NA |
| ACP11 | Paediatric | Relapsing ACP, initial complete resection, followed by recurrence 4 years later, which was treated with proton therapy. A further recurrence after a further 4 years showed histologically malignant features (see text) and rapid regrowth (<4 months) after apparent complete resection | 1 x primary 1 x most recent recurrence | *CTNNB1* G34R |
| **Non- recurrent ACP cases:** | | |  |  |
| ACP12 | Paediatric | Initial subtotal resection. No recurrence up to 8 years later when transferred to another hospital | 1 x primary | CTNNB1 S37C |
| ACP13 | Paediatric | Initial resection and radiotherapy. No recurrence after 10 years of follow up | 1 x primary | CTNNB1 S37C |
| ACP14 | Paediatric | Initial subtotal resection. No recurrence after 10 years of follow up | 1 x primary |  |
| **Recurrent PCP cases** |  |  |  |  |
| PCP1 | Adult | Recurrence 3 years after gross total resection | 1 x primary, 1x recurrence | BRAF V600E |
| PCP2 | Adult | Recurrence 6 years after subtotal resection | 2 x recurrence | *BRAF* V600E |
| PCP3 | Adult | Recurrence 3 years after subtotal resection | 1 x primary, 1x recurrence | NA |
| PCP4 | Adult | Recurrence 7 years after subtotal resection | 1 x recurrence | NA |

NA: not available.
